# Public evaluation of food safety: Changes and influencing factors from the perspective of social governance — Evidence from CSS data (2006–2023)

**DOI:** 10.1371/journal.pone.0362408

**Published:** 2026-11-12

**Authors:** Han Lin

**Affiliations:** School of Law and Social Work (School of Intellectual Property), Dongguan University of Technology, Dongguan, Guangdong, China; Iwate University: Iwate Daigaku, JAPAN

## Abstract

This study used descriptive analysis and multinomial logistic regression on seven‑wave Chinese Social Survey (CSS) data spanning 2006‑2023 to systematically examine long‑term trends and determinants of public food‑safety evaluation. Public food‑safety evaluation exhibited a phased “rise‑fall‑rise” mean score pattern across the study period. Level of social security showed a consistent significant positive correlation with food‑safety evaluation in all seven survey years (*p* < 0.001) with fluctuating‑yet‑growing effect sizes; for the “very safe vs. very unsafe” contrast, the odds ratio fell from 2.056 (2006) then rebounded to 2.201 (2023). In year‑stratified models, educational attainment had a stable negative association with food‑safety evaluation, whereas control‑variable effects including gender and age varied across survey waves. Statistically significant survey‑year‑by‑social‑security‑level interactions for most years indicated temporal fluctuation in the spill‑over effect of social governance on food‑safety evaluation. Model diagnostics revealed nearly all variance inflation factors (VIF) below 1.780, demonstrating no multicollinearity and robust conclusions. Comprehensive countermeasures — building a socially secure atmosphere, establishing food‑risk prevention and emergency‑response governance frameworks, and strengthening institutional trust to advance social‑governance performance — should be adopted to improve public food‑safety perceptions.

## Introduction

Social security shapes the effectiveness of social governance, and social governance in turn acts upon social security and development. As a vital component of social security, food safety has gained growing public attention. Food‑safety evaluation not only determines people’s quality of life and subjective wellbeing but also constitutes a key indicator for measuring social governance performance. Food‑safety issues do not exist in isolation; they are closely intertwined with trust and other social‑governance‑related elements. Trust constitutes the foundation of daily interactions [1] and the cornerstone of societal operation [2]. Without trust, society would slip into fragmentation, because nearly all human relationships are built upon interpersonal trust [3]. Production, processing, circulation and consumption of food all embody social interactions and cooperation, rendering trust particularly critical within the food‑safety domain. Public trust in food producers, processors, sellers and regulatory authorities directly shapes purchasing decisions and consumption experiences, and consumption experiences in turn mould public evaluations of food safety. Where trust erodes, market order deteriorates; consumers become anxious regarding food consumption and health risks, and social governance faces severe trust crises [4].

Globally, food‑safety challenges remain prominent. According to estimates from the World Health Organization (WHO), approximately 600 million people worldwide (nearly one in ten) fall ill annually from consuming contaminated food. This burden results in 33 million disability‑adjusted life‑years (DALYs) lost and 420 000 deaths each year, with low and middle‑income countries bearing the heaviest impacts [5]. Foodborne diseases can trigger over 200 distinct illnesses ranging from diarrhoea to cancer, in some cases causing permanent disability or death. The WHO Global Food Safety Strategy 2022‑2030 explicitly states that access to safe, nutritious and wholesome food represents a fundamental human right, yet large community segments cannot realise this right [6]. The Codex Alimentarius Commission (CODEX), the central global body for food‑safety standard‑setting, has formulated general principles and operational codes that provide important references for national food‑safety governance worldwide [7]. Within China, food safety attracts substantial public concern. The 2008 melamine‑tainted milk powder incident marked a landmark turning‑point in contemporary Chinese food‑safety history: approximately 300 000 infants and young children suffered poisoning after consuming melamine‑adulterated milk powder, with at least six infant fatalities [8]. Beyond massive health harm, this event severely undermined public trust in food‑safety regulatory systems and delivered important lessons for social governance. China has since continuously strengthened its food‑safety regulatory framework [9]. Nevertheless, despite improved regulatory systems, food‑safety risks persist, and public concerns over food safety are far from eliminated [10].

Existing Chinese scholarship on food safety focuses primarily on food safety supervision [11], construction of food safety standard systems [12], establishment of food safety guarantee systems [13], and public food safety perceptions [14]. Internationally, subjective public evaluations of food safety also receive widespread scholarly attention, with two dominant research strands. First, cross‑national comparative studies exploring disparities in food‑safety evaluation. Djekic et al. (2022) conducted a nine‑nation survey and found that consumers prioritise food hygiene as the most influential factor shaping food‑safety evaluations; animal‑derived foods receive the lowest ratings, and country of origin exerts the strongest influence on assessments [15]. Tonda et al. (2023) applied machine learning methods to consumer data across seven nations and demonstrated that consumer food‑safety evaluations are mainly explained by food knowledge levels rather than income and other factors [16]. Bogueva (2024) further illustrated substantial cross‑regional variation in public food safety perceptions, with distinct consumer attitudes observed across Africa, Asia, Europe and the Americas [17]. Second, research examining determinants and mechanisms underlying food‑safety evaluation. Tomasevic et al. (2023) found that the COVID‑19 pandemic substantially altered public food‑safety evaluations in a survey covering 15 Eurasian nations; European consumers reported higher evaluations than Asian consumers, and consumers displayed highest trust toward food scientists and certified food producers [18]. Soon‑Sinclair et al. (2023) studying five Southeast Asian countries reported that consumer food‑safety evaluations are jointly affected by media information, personal social networks, credible organisational information sources, and personal lived experiences [19]. A systematic review commissioned by the European Food Safety Authority conducted by Jaskiewicz K et al. (2023) indicated that consumer food‑safety evaluations do not always correspond to objective risk levels, and certified‑labelled food products can effectively improve consumer assessments [20]. A systematic review by Suhaimi et al. (2024) identified four core categories of factors shaping consumer food safety assessments: product attributes, pre‑existing food safety perceptions, trust in food systems, and consumer socio‑economic characteristics [21]. Reitano M et al. (2024) performed a systematic review showing that consumer trust‑based food‑safety evaluations decline amid supply‑chain complexity, and blockchain traceability technologies hold potential for enhancing trust assessments [22]. Moreover, food‑safety evaluations are shaped by real‑world food contamination and household food‑handling behaviours: Serhan et al. (2024) presented empirical evidence on microbial hazards within retail‑meat environments; Karam et al. (2024) directly analysed associations between household food safety practices and consumer behaviours; Merhi et al. (2026) performed exposure assessments that underscored the importance of chemical‑contaminant monitoring for boosting public confidence [23–25].

Existing studies provide valuable cross‑national perspectives and factor‑identification foundations, yet most rely on cross‑sectional surveys. Long‑term systematic investigations into temporal shifts in public food‑safety evaluation remain scarce, and social‑governance‑related factors represented by the overall level of social security have not been incorporated within localized Chinese analyses. Food‑safety evaluation is essentially a subjective trust judgement formed by consumers based on food quality, personal experience, risk perception, and multiple social‑governance‑related factors. How has this subjective trust evolved in Chinese society over nearly two decades? Which factors exert significant influences? Motivated by these research questions, we carried out this study.

## Materials and methods

### Data source and analytical framework

This study draws upon the Chinese Social Survey (CSS), led by the Chinese Academy of Social Sciences. CSS adopts a multi‑stage complex sampling design. Counties/districts across mainland China serve as primary sampling units (PSUs), implicitly stratified according to economic development, population composition and educational indicators. Probability‑proportional‑to‑size (PPS) sampling selects PSUs, followed by sequential sampling of neighbourhood/village committees, households and individual respondents [26–28]. The survey covers more than 30 provinces/autonomous regions in China and targets citizens aged 18‑80 years old. This study selected samples possessing complete food‑safety‑evaluation variables across seven survey waves: 2006, 2008, 2013, 2015, 2017, 2021 and 2023.

For variable selection, this study combined theoretical guidance with data availability. Included variables are consistently measured across CSS waves with low missing rates, ensuring cross‑year comparability. Several potentially important confounding variables (e.g., household income, urban‑rural residence, geographic region, occupation) could not be incorporated due to data limitations. The analytical framework aims to test the effect of the level of social security upon food‑safety evaluation, explore the moderating role of survey year for this core relationship, while adjusting for demographic covariates including gender, age, marital status, political affiliation, and educational attainment (Fig 1).

**Fig 1:**
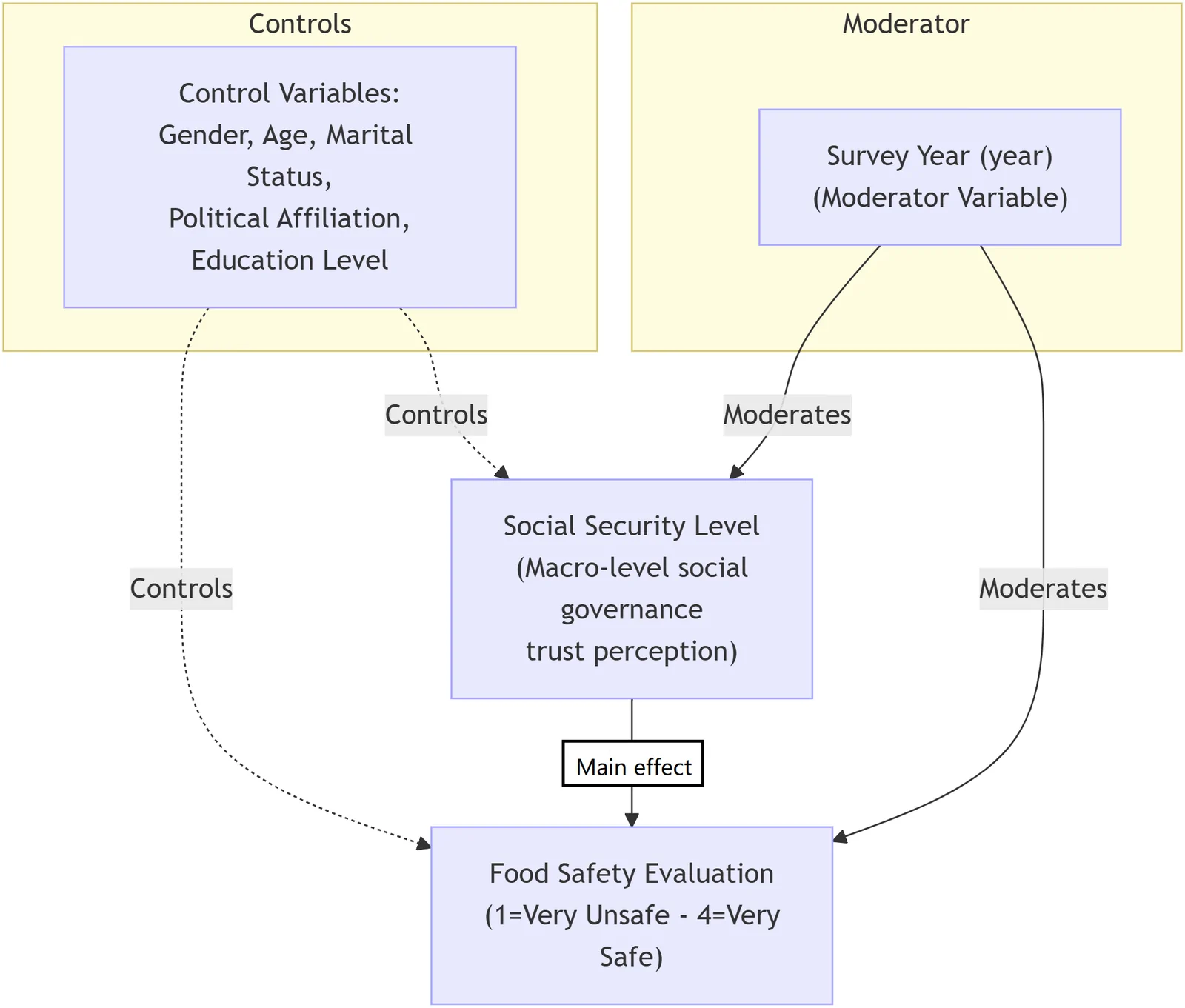
Analytical framework diagram.

### Dependent variable processing

The dependent variable is the public’s subjective evaluation of food safety, derived from the CSS survey item: “How would you rate the current level of food safety?”. Response options: 1 = Very unsafe, 2 = Not very safe, 3 = Relatively safe, 4 = Very safe. Responses such as “Don’t know” or “Hard to say” are treated as missing values [26]. Food‑safety evaluation represents subjective consumer risk perceptions regarding health hazards within food consumption, alongside trust judgements concerning the effectiveness of supervision across food production, processing and circulation links. Higher scores correspond to greater public trust in food safety.

Table 1 reports raw four‑category frequency distributions of public food‑safety evaluations across the seven survey years from 2006 to 2023. Valid sample size is smaller for 2015 (n = 3 381), because CSS adopted split‑form A/B questionnaires in that year and the food‑safety item only appeared on Form A [26]. Longitudinal descriptive results show that respondents selecting “Relatively safe” (score 3) constituted the largest group in all years. Its proportion declined notably in 2013 (38.3%) and 2015 (36.0%), then gradually rebounding, reaching 56.1% in 2021 and maintaining 52.7% in 2023. Correspondingly, respondents selecting “Very safe” (score 4) remained low from 2008‑2015 (below 10% for all waves except 2008 at 13.8%), but rose sharply in 2021 (20.1%) and 2023 (21.2%), indicating substantial growth in top‑tier public recognition of food safety in recent years. Conversely, combined proportions holding negative evaluations (score 1 + score 2) remained high above 50% during 2013‑2017, peaking at 57.3% in 2015. Negative‑evaluation shares subsequently dropped to 23.8% (2021) and 26.1% (2023), signalling overall recovery in public confidence regarding food safety.

**Table 1:** Raw category frequency distributions for food‑safety evaluation across survey years.

Year	Very unsafe (1) N (%)	Not very safe (2) N (%)	Relatively safe (3) N (%)	Very safe (4) N (%)	Total N
2006	526 (7.8%)	2,065 (30.5%)	3,277 (48.5%)	892 (13.2%)	6,760
2008	336 (4.8%)	1,935 (27.7%)	3,747 (53.7%)	960 (13.8%)	6,978
2013	1,487 (15.0%)	3,821 (38.6%)	3,790 (38.3%)	805 (8.1%)	9,903
2015	524 (15.5%)	1,414 (41.8%)	1,217 (36.0%)	226 (6.7%)	3,381
2017	1,471 (15.0%)	3,732 (38.0%)	3,577 (36.5%)	1,028 (10.5%)	9,808
2021	354 (3.6%)	1,989 (20.2%)	5,512 (56.1%)	1,974 (20.1%)	9,829
2023	349 (5.6%)	1,283 (20.5%)	3,306 (52.7%)	1,331 (21.2%)	6,269

Note: Percentages are calculated within each survey-year sample.

Table 2 presents descriptive statistics including mean and standard deviation for food‑safety‑evaluation scores for each survey year between 2006‑2023. Mean scores reveal clear phased fluctuations in subjective public food‑safety assessments. The mean rose modestly from 2.671 (2006) to 2.764 (2008). Notably, the 2008 CSS fieldwork concluded prior to the large‑scale outbreak of the melamine‑tainted‑milk‑powder incident; this 0.093‑point increase therefore reflects natural growth in public confidence unaffected by this major safety crisis [8]. Subsequent substantial declines in mean food‑safety scores in CSS2013 and CSS2015 (means < 2.40) closely correspond to cumulative impacts of successive food‑safety incidents. Means then recovered gradually: 2.424 in 2017, peak value 2.926 in 2021, with a slight decrease to 2.896 in 2023 while maintaining a high level. This trend demonstrates that following the mid‑2010s trough in public food‑safety evaluations, assessments have improved markedly in recent years.

**Table 2:** Mean, standard deviation and distribution statistics of food‑safety‑evaluation scores across survey years.

Statistic	2006	2008	2013	2015	2017	2021	2023
Valid observations	6760	6978	9903	3381	9808	9829	6269
Mean	2.671	2.764	2.395	2.339	2.424	2.926	2.896
Standard deviation	0.800	0.743	0.838	0.817	0.868	0.736	0.793
Median	2.682	2.769	2.408	2.349	2.429	2.951	3.000
Mode	3	3	2	2	2	3	3
Variance	0.640	0.552	0.702	0.668	0.754	0.542	0.629
Minimum	1	1	1	1	1	1	1
Maximum	4	4	4	4	4	4	4

### Independent variables, control-variables and hypotheses

The core independent variable in this study is the level of social security. It is constructed by summing multiple CSS social‑security‑perception items covering property security, personal physical safety, traffic safety, medical safety, labour‑related safety, personal‑information‑and‑privacy security, and environmental‑safety dimensions. Scores range 0‑50; higher scores indicate higher respondent evaluations of macro‑level social‑governance performance [26]. The theoretical rationale for including level of social security is that public trust in food safety does not exist in isolation; it is embedded within trust toward the overall social‑governance system. Good performance in domains such as social order, public safety and environmental governance typically generates higher institutional trust, which in turn reinforces public confidence in food‑safety regulatory systems [2].

Drawing upon existing scholarship on social governance (including trust‑related factors) [29–31], this study controls five demographic variables: gender, age, marital status, political affiliation and educational attainment. Survey year functions as the moderating variable. Variable coding definitions are summarised in Table 3.

**Table 3:** Variable definitions and coding schemes.

Variable	Definition and coding
Food‑safety evaluation (dependent variable)	1 = Very unsafe, 2 = Not very safe, 3 = Relatively safe, 4 = Very safe (ordinal categorical variable)
Level of social security (independent variable)	Summed score across social security perception items (0–50), continuous variable; higher scores represent more positive assessments of social security
Gender	1 = Male, 0 = Female (reference group: Female)
Age	Respondent’s actual age, continuous variable (18–80 years old)
Marital status	1 = Spouse present (first marriage spouse / remarried after divorce / remarried after bereavement); 0 = No spouse (unmarried / cohabiting / divorced without remarriage / widowed without remarriage) (reference group: No spouse)
Political affiliation	1 = Political identity holder (Communist Party of China member / Communist Youth League member / democratic party member); 0 = Mass (reference group: Mass)
Educational attainment	1 = No formal education, 2 = Primary school, 3 = Junior high school, 4 = High school / vocational high school / technical school / secondary specialised school, 5 = Junior college, 6 = Bachelor’s degree, 7 = Postgraduate and above (treated as continuous variable)
Survey year	1 = 2006, 2 = 2008, 3 = 2013, 4 = 2015, 5 = 2017, 6 = 2021, 7 = 2023 (categorical / dummy variable; reference category = 2023)

Based upon the theoretical framework outlined above, the following hypotheses are proposed:

H1: The level of social security is positively associated with food‑safety evaluation; higher public evaluations of macro‑level social governance correspond to higher evaluations of overall social security and higher trust levels regarding food safety.

H2: Males, respondents with a spouse, and respondents holding political identities report higher food‑safety evaluations.

H3: Age and educational attainment exhibit negative associations with food‑safety evaluation: older respondents and respondents with higher educational attainment hold lower food‑safety evaluations.

H4: Survey year exerts a significant moderating effect upon the relationship between level of social security and food‑safety evaluation (taking 2023 as reference, moderating effects differ across other survey years).

The theoretical grounding for these hypotheses derives from social‑governance‑and‑trust literature: women, older adults and higher‑educated individuals are generally more sensitive toward diverse social risks and demonstrate lower trust and stricter evaluative standards [29,30]. Institutional‑trust theory indicates that social‑integration functions associated with political identity and marital status may strengthen individual identification with governance systems and thereby enhance trust assessments within specific regulatory domains [2,3]. Year‑based moderation originates from temporal shifts in social‑governance performance and public perceptions.

### Model specification

The dependent variable is four‑category food‑safety evaluation (ranging from 1 = Very unsafe to 4 = Very safe). If dichotomised, binary logistic regression discards substantial information and cannot distinguish between “Relatively safe” and “Very safe”. Comparative analyses also show that Nagelkerke pseudo‑R^2^ values for binary‑logistic‑regression models are generally lower than those for multinomial logistic regression. Ordinal logistic regression requires satisfaction of the proportional‑odds assumption, meaning predictor coefficients for cumulative logits remain constant across cut‑points. We performed parallel‑lines tests for each yearly dataset to verify this assumption; results appear in Table 4.

**Table 4:** Parallel‑lines test results (proportional‑odds‑assumption test) for ordinal logistic regression by survey year.

Year	Chi‑square	df	p‑value	Assumption satisfied
2006	41.291	22	0.008	No
2008	14.422	12	0.275	Yes
2013	155.080	12	<0.001	No
2015	73.208	12	<0.001	No
2017	608.355	12	<0.001	No
2021	675.234	12	<0.001	No
2023	389.134	12	<0.001	No

As shown in Table 4, the proportional‑odds assumption is rejected for six out of seven years (*p* < 0.05), with only 2008 satisfying this requirement. Therefore, multinomial logistic regression is adopted for this study [32]. Let Y denote the four‑category dependent variable of food‑safety evaluation (1 = Very unsafe, 2 = Not very safe, 3 = Relatively safe, 4 = Very safe). In the year-specific models, “Very unsafe” (Y = 1) serves as reference category. The log‑odds ratio for category a versus reference category 1 is specified as:


$$\begin{array}{c} \text{ln}\left(\frac{P(Y=a)}{P(Y=\text{1})}\right)=\alpha_{a}+\sum_{p=\text{1}}^{P}\beta_{ap}X_{p},\;a=\text{2},\text{3},\text{4} \end{array}$$

where *α_a_* is the intercept parameter between category *a* and the reference category, *β_ap_* is the partial regression coefficient of the *p*‑th predictor variable on category *a* (relative to the reference category), and *X_p_* is the *p*‑th predictor variable. The odds ratio (OR) for each predictor variable is exp(*β_ap_*), which represents the fold change in the odds of the dependent variable falling into category a relative to the reference category for each one‑unit increase in the predictor variable [32]. In the pooled data analysis, the model further included the interaction term between survey year (year) and social security level to test whether the effect of social security level differed significantly across years [33].

## Results

### Separate‑wave multinomial logistic‑regression analyses

#### Model‑fit information.

Separate multinomial logistic‑regression models were estimated for each of the seven survey years. The four‑category food‑safety‑evaluation dependent variable uses “Very unsafe” (1.00) as reference category. Valid sample sizes are smaller than samples reported in Table 1 and Table 2, as the social‑security‑level scale items only appeared within specific questionnaire forms in individual survey years, and missing values exist among control variables. Core independent variable: level of social security; control variables include gender, age, marital status, political affiliation and educational attainment. Year‑specific model‑fit statistics are summarised in Table 5.

**Table 5:** Year‑specific multinomial‑logistic‑regression model‑fit statistics.

Year	Valid sample (n)	Model χ²	df	p value	Nagelkerke R²
2006	6,757	1,830.650	18	<0.001	0.262
2008	6,975	1,323.477	18	<0.001	0.194
2013	9,809	2,359.687	18	<0.001	0.234
2015	3,230	717.982	18	<0.001	0.218
2017	9,789	2,519.330	18	<0.001	0.247
2021	9,816	4,538.486	18	<0.001	0.417
2023	6,263	3,268.221	18	<0.001	0.452

Likelihood‑ratio χ^2^ tests were statistically significant (*p* < 0.001) across all models, indicating superior fit compared with intercept‑only null models. Nagelkerke pseudo‑R^2^ fluctuated: 0.262 (2006), 0.194 (2008), then fluctuated further before rising to 0.452 in 2023. This demonstrated fluctuating but overall increasing explanatory power over time, with substantial improvements observed for the most recent three survey waves (2021‑2023). Although the 2015 model had relatively small valid sample size (n = 3 230), its model χ^2^ (717.982) and pseudo‑R^2^ (0.218) remained within reasonable ranges.

#### Cross‑year effects of the core independent variable: level of social security.

Level of social security constituted this study’s core independent variable. Regression coefficients, odds ratios (OR), standard errors, 95% confidence intervals, CI width and Wald χ^2^ statistics for each survey year under the “Very safe vs. Very unsafe” contrast are presented in Table 6. Results showed that level of social security exhibited significant positive associations with food‑safety evaluation in all seven survey years (*p* < 0.001), demonstrating that better social‑governance performance and higher public perceptions of overall social security correspond to higher trust levels regarding food safety [34].

**Table 6:** Year‑specific effects of level of social security on food‑safety evaluation and parameter‑estimation precision (“Very safe” vs. “Very unsafe”).

Year	B coefficient	SE	Wald χ²	p‑value	OR	95% CI (OR)	CI width
2006	0.721	0.025	864.328	<0.001	2.056	[1.959, 2.157]	0.198
2008	0.552	0.022	641.357	<0.001	1.737	[1.664, 1.813]	0.149
2013	0.376	0.012	914.452	<0.001	1.457	[1.422, 1.493]	0.071
2015	0.379	0.022	292.789	<0.001	1.461	[1.399, 1.526]	0.127
2017	0.458	0.013	1,225.529	<0.001	1.581	[1.541, 1.622]	0.081
2021	0.746	0.019	1587.531	<0.001	2.109	[2.033, 2.187]	0.154
2023	0.789	0.021	1,389.005	<0.001	2.201	[2.112, 2.294]	0.182

Note: *B*, regression coefficient; SE, standard error; Wald χ^2^, Wald chi‑square statistic; OR, odds ratio; CI, confidence interval.

Regarding temporal variation in effect sizes: the B coefficient for level of social security for “Very safe” relative to “Very unsafe” declined continuously from 0.721 (2006), followed by sustained increases post‑2013 to reach 0.789 in 2023. Odds ratios similarly showed fluctuating patterns: starting from 2.056 (2006), decreasing to minimum value 1.457 in 2013, then gradually rising to 2.201 in 2023. This trend indicated that positive effects exerted by level of social security upon food‑safety evaluation fluctuated over time and entered an accelerated‑growth phase after 2013. Years 2015 (OR = 1.461, 95% CI: 1.399‑1.526) and 2017 (OR = 1.581, 95% CI: 1.541‑1.622) marked the onset of rapid effect‑size growth, with peak values observed in 2021 (OR = 2.109) and 2023 (OR = 2.201).

We further assessed precision of parameter estimates. Standard errors for level of social security ranged 0.012‑0.025 across years; 95%‑confidence‑interval width (upper‑bound minus lower‑bound) ranged 0.071‑0.198, indicating reasonably precise parameter estimates. Across all seven years, Wald χ^2^ statistics for level of social security substantially exceeded critical thresholds (minimum = 292.789; maximum = 1 389.005), and all associated p‑values were below 0.001. These findings showed that core‑predictor effects were not only statistically significant but also were estimated with high precision, and were not materially affected by sample fluctuations or model specification choices [32].

#### Cross‑year effects of control variables.

Direction and statistical significance of control‑variable effects for the “Very safe vs. Very unsafe” contrast for each survey year are summarised in Table 7.

**Table 7:** Direction and significance of control‑variable effects by survey year (“Very safe” vs. “Very unsafe”).

Variable	2006	2008	2013	2015	2017	2021	2023
Gender (Male = 1)	−**	−**	−**	−**	−***	−**	—
Age	−**	—	—	—	+***	−*	—
Marital status (Spouse = 1)	—	—	—	—	+**	—	+**
Political affiliation (Identity holder = 1)	—	—	—	—	−**	—	−**
Educational attainment	−***	−***	−***	−***	−***	−***	−***

Note: + = positive effect; − = negative effect; *** *p* < 0.001; ** *p* < 0.01; * *p* < 0.05; — = not significant (*p* ≥ 0.05).

Educational attainment represented the sole variable maintaining high statistical significance (*p* < 0.001) with consistent negative directions across all years, showing that respondents with higher educational attainment held more prudent food‑safety evaluations [29,31]. Gender showed a negative direction in most years (males’ evaluations were lower than females’), but it was not significant in 2023. Age effects fluctuated substantially: significantly negative in 2006, significantly positive in 2017, significantly negative again in 2021; nonsignificant or marginally significant for remaining years. Such patterns may relate to differing phases of food‑safety‑crisis shocks and recovery. Marital status attained statistical significance only in 2017 (positive) and 2023 (positive). Political affiliation attained statistical significance only in 2017 (negative) and 2023 (negative); respondents holding political identities reported lower evaluations, suggesting they applied stricter assessment criteria [11]. Overall, educational attainment was the most robust predictor, while effect directions for age, marital status and political affiliation fluctuated across survey years.

### Pooled‑data interaction‑effect analysis

#### Model specification.

To formally test interaction effects between survey year and level of social security, data from all seven survey waves were pooled, and a multinomial logistic‑regression model incorporating year × level‑of‑social‑security interaction terms was constructed. The 2023 survey wave serves as reference group. The four‑category food‑safety‑evaluation dependent variable uses “Very safe” as reference category. Covariates incorporated are gender, marital status, political affiliation, age, educational attainment and main effects for survey year. Valid pooled sample size = 52,642.

#### Model fit.

Likelihood‑ratio χ^2^ test result for pooled‑data model: χ^2^ = 20,178.163 (df = 54, *p* < 0.001); Nagelkerke pseudo‑R^2^ = 0.349, Cox‑Snell R^2^ = 0.318, McFadden R^2^ = 0.158. The pooled‑data model fitted data well; incorporated variables exerted substantial explanatory power over food‑safety evaluation outcomes.

#### Interaction‑effect results (contrast: Relatively safe vs. Very safe).

Table 8 presents regression coefficients for year‑by‑social‑security‑level interaction terms. Interaction coefficients for 2006‑2017 relative to the 2023 reference group were statistically significant (*p* < 0.001), meaning social‑security‑level effects on food‑safety evaluation differed across these survey years. The 2021 interaction term was non‑significant (*B* = 0.012, *Wald* = 0.398, *p* = 0.528), showing its effect pattern no longer differed from 2023 and had lost the distinct year‑specific features seen in 2013‑2015, possibly indicating reconstructed public trust structures. Note that Table 8 only reports coefficients relative to 2023; the 2023 main effect is incorporated in the model intercept and parameters and is not listed as an interaction term.

**Table 8:** Interaction effects between survey year and level of social security (“Relatively safe” vs. “Very safe”).

Variable	B coefficient	SE	Wald χ^2^	p value
year2006 × Level of social security	0.208	0.022	90.178	<0.001
year2008 × Level of social security	0.331	0.018	329.750	<0.001
year2013 × Level of social security	0.389	0.017	533.795	<0.001
year2015 × Level of social security	0.376	0.021	333.442	<0.001
year2017 × Level of social security	0.282	0.018	240.335	<0.001
year2021 × Level of social security	0.012	0.019	0.398	0.528
Gender (Female vs. Male)	0.053	0.032	2.757	0.097
Marital status (No spouse vs. Spouse)	−0.066	0.043	2.384	0.123
Political affiliation (No identity vs. Identity holder)	0.004	0.052	0.007	0.933
Age	−0.004	0.001	9.313	0.002
Educational attainment	0.260	0.015	289.567	<0.001

Note: The reference category for the dependent variable is “Very safe” (4.00); the main effect of survey year takes 2023 as the reference group. Table 8 only lists interaction coefficients contrasting each survey year against 2023.

Interaction effect sizes showed clear phased shifts: moderate magnitudes in 2006 (*B* = 0.208, *Wald* = 90.178) and 2008 (*B* = 0.331, *Wald* = 329.750), peak values in 2013 (*B* = 0.389, *Wald* = 533.795) and 2015 (*B* = 0.376, *Wald* = 333.442) — coinciding with the 2013‑2015 trough in mean food‑safety evaluations — followed by a decline in 2017 (*B* = 0.282, *Wald* = 240.335) and non‑significance for 2021.

This yielded the “synchronous resonance” pattern: interaction coefficients for social‑security level were largest during the 2013‑2015 low‑point of food‑safety evaluations (*B* = 0.376‑0.389), yet became smallest or non‑significant when evaluations rebounded in 2021‑2023 (2021: *B* = 0.012). This apparent counter‑intuitive pattern stems from the reference‑year setup: Table 8 gives coefficients relative to 2023 rather than per‑year total effects. The 2023 main effect of social‑security level was B = −0.528 (*Wald* = 1264.161, *p* < 0.001). Summing main and interaction terms gives combined effects: the absolute combined‑effect magnitude for 2021 (−0.528 + 0.012 = −0.516) exceeded that for 2013 (−0.528 + 0.389 = −0.139). Thus, social‑security level still exerted substantial leverage to shift respondents toward higher evaluation categories when food‑safety evaluations were high, even though the 2021 interaction itself was non‑significant and its combined effect stemmed mostly from the persistent 2023 main effect. This pattern pointed to systematic co‑movement between macro‑governance trust from social‑security perceptions and domain‑specific food‑safety public trust [2,34]; this spill‑over effect strengthened over time, suggesting food‑safety trust was increasingly embedded within assessments of overall governance performance.

Wald statistics for the yearly interaction terms rose from 90.178 (2006) to a peak of 533.795 (2013), then fell to 0.398 (2021). The 2017 Wald value (240.335) quantified its difference versus 2023; the negligible, non‑significant 2021 Wald contrasted with the higher 2013 and 2015 values, which signalled more reliable interaction‑effect estimates for those two years.

It should be noted that the positive B coefficient for educational attainment in Table 8 (*B* = 0.260) corresponds to the “Relatively safe vs. Very safe” contrast, whereas Table 7 reports results for “Very safe vs. Very unsafe”. The former showed that higher‑education respondents were more likely to shift evaluations from “Very unsafe” toward “Relatively safe”; the latter simultaneously showed they were less inclined to give extreme‑positive (“Very safe”) assessments. These divergent outcomes across cut‑points do not constitute statistical contradictions within multinomial‑logistic‑regression frameworks.

### Model diagnostics and robustness checks

#### Assessment of parameter‑estimation precision.

Classification‑accuracy metrics for the multinomial logistic regression models across survey years were summarised in Table 9. Overall predictive accuracy ranged from 47.0% to 67.3%, well above the 25% expected under random classification [32]. Category‑specific performance was strongest for the “Relatively safe” (score 3) category (51.4%‑94.4%), consistent with its largest respondent share each year (36.0%‑56.1%). By contrast, the extreme‑response categories “Very unsafe” (score 1; 0.3%‑10.3%) and “Very safe” (score 4; 5.3%‑70.8%) yielded comparatively low predictive accuracy, an inherent limitation stemming from their smaller sample sizes [35]. Notably, “Very safe” predictive accuracy rose to 66.8% in 2021 and 70.8% in 2023, far exceeding earlier‑year values (18.9% for 2006, 7.6% for 2008, 5.6% for 2013). This improvement aligned with the growing share of “Very safe” responses (20.1% in 2021, 21.2% in 2023), as larger group sizes enhanced the model’s ability to detect outcome patterns. The full pooled‑data model (including year × level‑of‑social‑security interaction terms) attained 56.0% overall accuracy and 79.3% accuracy for the “Relatively safe” category, further confirming cross‑sample predictive stability.

**Table 9:** Prediction accuracy (%) of multinomial logistic regression models by survey year and for pooled data.

Year	Very unsafe (1)	Not very safe (2)	Relatively safe (3)	Very safe (4)	Overall accuracy
2006	5.5	34.5	84.5	18.9	54.4
2008	7.7	14.2	94.4	7.6	56.1
2013	10.3	62.1	62.6	5.6	49.9
2015	3.9	66.8	51.4	5.3	47.0
2017	4.2	66.6	54.0	29.1	48.7
2021	0.3	8.3	93.2	66.8	67.3
2023	2.9	7.9	91.5	70.8	65.1
Pooled data (with interaction terms)	5.2	44.1	79.3	38.9	56.0

Note: Predictive results for the pooled data are derived from the full multinomial logistic regression model incorporating year × level‑of‑social‑security interaction terms.

#### Multicollinearity diagnostics.

Variance inflation factors (VIF) were computed for both year‑stratified and pooled‑data models using SPSS statistical software [36,37]. Year‑specific VIF values are summarised in Table 10. All VIF values remained well below conventional critical thresholds of 10 (and stricter threshold 5), ranging 1.003‑1.780. This indicated no severe multicollinearity; all independent variables could safely enter regression models simultaneously [37].

**Table 10:** Year‑specific variance‑inflation‑factor (VIF) values for model variables.

Variable	2006	2008	2013	2015	2017	2021	2023
Level of social security	1.033	1.030	1.117	1.025	1.017	1.003	1.009
Gender	1.038	1.028	1.053	1.032	1.043	1.037	1.026
Age	1.177	1.242	1.329	1.460	1.456	1.517	1.559
Marital status	1.080	1.150	1.111	1.283	1.170	1.194	1.208
Political affiliation	1.189	1.223	1.318	1.425	1.339	1.466	1.282
Educational attainment	1.303	1.347	1.606	1.665	1.626	1.780	1.656

Diagnostic results for the pooled model (containing year dummies and their interactions with level of social security) are presented in Table 11. The VIF values for the routine control variables and for level of social security were all below 10, indicating no multicollinearity concerns. The year dummies and interaction terms exhibited relatively high VIF values (36–60) due to their construction, which was an inherent phenomenon in regression models that include interaction terms [38] and did not affect the unbiasedness of parameter estimation. Centering the level of social security could effectively reduce this multicollinearity, ensuring that the model estimates remained robust and reliable.

**Table 11:** Variance inflation factors (VIF) for the pooled model (containing year dummies and interaction terms).

Variable category	Variable name	VIF range	Assessment
Control variables	Age, gender, marital status, political affiliation, educational attainment	1.133–3.155	No multicollinearity
Core variable	Level of social security	8.206	Acceptable (<10)
Year dummies	year2006 – year2021	37.147–60.566	Inherent collinearity^a^
Interaction terms	Year dummies × level of social security	36.887–55.492	Inherent collinearity^a^

Note: ^a^ The high VIF values for year dummies and interaction terms arise from the multiplicative collinearity inherent in constructing interaction terms; this does not affect the unbiasedness of coefficient estimates and can be mitigated by centering the level of social security.

#### Variable‑selection strategy.

For variable‑selection, this study adopts purposeful‑selection stepwise‑regression strategies described in Bursac et al. (2008) [39]. Likelihood‑ratio tests serve as criteria for variable entry and removal; entry threshold *p* < 0.05, removal threshold *p* > 0.10. Before model inclusion, univariate screening retained variables with *p* < 0.25 for multivariate‑model entry [39]. In practice, gender, age, marital status, political affiliation, educational attainment and level of social security all passed univariate screening and entered final multinomial‑logistic‑regression models. This variable‑selection workflow maintains parsimony while avoiding omission of potentially important predictor variables.

#### Data sparsity concerns.

Case‑processing summaries showed that the dependent variable yielded only one observed value across numerous subgroups in each survey year: 76.9% (2006), 59.2% (2008), 77.6% (2013), 87.9% (2015), 76.1% (2017), 78.7% (2021), and 86.6% (2023). This phenomenon warranted attention for multinomial logistic regression, since it pointed to substantial observation sparsity across many independent‑variable combinations, which might impair parameter‑estimate stability [32]. Nevertheless, all year‑specific models converged successfully, and likelihood‑ratio chi‑square tests attained statistical significance (*p* < 0.001). This indicated that, notwithstanding sparse‑data conditions, the models effectively detected significant associations between core predictors and the dependent variable. Moreover, level of social security remained highly statistically significant (*p* < 0.001) in every year and displayed a fluctuating‑increasing effect‑size trend, further showing that core conclusions were only minimally influenced by data sparsity.

#### Multiple‑comparison adjustment.

This study performed numerous statistical tests across seven survey waves and multiple predictors, raising multiple‑comparison concerns. Bonferroni correction was applied to our key predictor, level of social security [38]. Twenty‑one separate tests were run for this variable in year‑specific models (7 years × 3 outcome contrasts: Not very safe vs. Very unsafe; Relatively safe vs. Very unsafe; Very safe vs. Very unsafe). All *p*‑values were below 0.001. Even under the most‑conservative Bonferroni correction (adjusted *α* = 0.05 / 21 ≈ 0.00238), all results remained statistically significant (*p* < 0.001 < 0.00238), demonstrating high statistical robustness for the level‑of‑social‑security effect against multiple‑comparison bias. (Note: Tests for year‑interaction terms in the pooled‑data model were excluded from this correction; the non‑significant 2021 interaction finding is reported and interpreted in Section 3.2.) Control‑variable results reaching only marginal significance in individual years (*p* = 0.05‑0.10) are treated as suggestive findings requiring future validation.

## Discussion

Drawing upon descriptive analysis and multinomial‑logistic‑regression analyses of seven‑wave CSS data from 2006‑2023, this study systematically investigates long‑term trends and influencing factors for public food‑safety evaluation. Main findings are summarised below.

Public food‑safety evaluation exhibited a long‑term “rise‑fall‑rise” phased‑fluctuation pattern. Mean scores rose modestly 2006‑2008, dropped to a trough during 2013‑2015, recovered gradually afterwards, peaked in 2021, and stabilised near 2.90 in 2023. This temporal pattern closely aligns with real‑world food‑safety‑crisis occurrences and national‑governance trajectories in China. The large‑scale melamine‑tainted‑milk‑powder crisis did not influence 2008 CSS fieldwork (which concluded before crisis escalation), yet cumulative crisis effects manifested clearly within 2013‑2015 survey data. Following revision and implementation of the Food Safety Law and continuous improvements within regulatory frameworks post‑2015, public confidence entered a recovery phase [9]. Notably, trust‑recovery cycles last approximately eight years, demonstrating that rebuilding food‑safety‑related trust represented a slow and difficult societal process.

Level of social security constituted the strongest and most robust positive predictor of food‑safety evaluation. It was significantly positively associated with food‑safety evaluation across all seven survey years (*p* < 0.001), with fluctuating yet growing effect sizes illustrated using the “Very safe vs. Very unsafe” contrast. This finding aligns with core propositions from social governance and trust theory: public trust regarding food safety does not emerge in isolation; it is embedded within broader trust toward overall social governance systems [2,34]. Higher public assessments of overall social security foster institutional trust that spills over into food safety domains [40]. Critically, significant year × level of social security interaction effects (*p* < 0.001) demonstrated that this spill‑over effect grew stronger with time. This suggested that food‑safety‑related trust had become increasingly embedded within public evaluations of macro‑level social governance performance.

In separate year‑by‑year models, educational attainment showed significant negative associations with food‑safety evaluation for every survey year. Respondents with higher educational attainment expressed more prudent food‑safety assessments. This finding aligns with theoretical expectations: individuals with higher educational attainment enjoy broader information access channels and apply stricter scrutiny to food safety standards [29,31]. By contrast, within pooled‑data interaction‑included models, the baseline main effect coefficient for educational attainment (reference year = 2023) is significantly positive. This divergence points toward substantial temporal heterogeneity for educational attainment effects. Hypothesised relationships for gender, age, marital status and political affiliation received incomplete empirical support. Gender effects exhibited directional differences across survey years and across different levels of food‑safety evaluation. In year‑specific separate models, gender showed a negative effect in most years (men reported lower evaluations than women). By contrast, in the pooled interaction model, the effect became positive for the “Relatively safe versus Very unsafe” comparison (*B* = 0.053). This directional shift indicated that gender‑related influences varied depending on the reference outcome category, and also suggested that such effects were unstable under certain model specifications. Age‑related effects shifted according to food safety crisis phases: older respondents demonstrated heightened risk perceptions (negative effects) during crisis outbreak periods yet might produce more positive evaluations driven by life experience during trust recovery phases. Social integration effects associated with marital status and political affiliation only generated food‑safety domain trust spillovers in specific survey years (2017, 2023), with weak and unstable overall magnitudes. Collectively these results demonstrated that control‑variable influences on food‑safety evaluation were not static; they were jointly moderated by macro social contexts and model reference categories and merited further validation in subsequent research.

Interpreted within international‑comparative perspectives, our findings correspond closely to the “institutional‑trust” dimension among four key consumer‑food‑safety‑evaluation factor categories identified by Suhaimi et al. (2024): product characteristics, pre‑existing perceptions, institutional trust, and socioeconomic consumer attributes [21]. Results also echo cross‑national Eurasian‑sample findings from Tomasevic et al. (2023): consumer trust in food‑safety systems strongly shapes subjective food‑safety assessments [18]. However, this study further reveals that social‑governance captured by level of social security generates dynamic “spill‑over effects” that grow in a fluctuating manner over time. These results supply new empirical evidence for understanding cumulative and context‑dependent characteristics of social‑governance effects. Meanwhile, the negative association between educational attainment and food‑safety evaluation reported herein resonates with cross‑national results from Tonda et al. (2023): consumer food‑safety assessments are largely explained by individual food‑knowledge levels [16].

Policy implications. Results point toward three policy‑relevant directions. First, growing spill‑over effects originating from level of social security indicate that improving public food‑safety perceptions cannot rely exclusively upon food‑safety‑supervision measures. Fostering an overall socially secure societal atmosphere and advancing comprehensive social‑governance initiatives including environmental‑quality improvements, although seemingly unrelated to food safety, can indirectly lift public food‑safety evaluations by strengthening institutional‑trust toward social‑governance systems [40,41]. Second, the approximately eight‑year trust‑recovery cycle highlights the necessity of constructing food‑risk‑prevention, early‑warning and emergency‑response governance architectures, to avoid sharp trust collapses triggered by major food‑safety incidents and shorten trust‑recovery durations [6,7]. Third, divergent effects for educational attainment, gender and political identity indicate value in differentiated risk‑communication governance strategies for distinct population subgroups: provide comprehensive scientific information for highly‑educated subgroups [23]; deliver practical food‑safety‑protection guidance targeted toward female consumers [24]; encourage citizens holding political identities to participate within food‑safety social‑supervision activities [25]. Domestic multi‑collaborative‑food‑safety‑governance scholarship supports these policy recommendations [42]. Top‑level thinking regarding diversified food‑supply‑system construction also delivers reference value for food‑safety‑domain social‑governance practice [43].

Study limitations. Several limitations of this study deserve attention when interpreting results. First, CSS constitutes repeated cross‑sectional survey data rather than true panel data tracking identical respondents across time. Reported associations represent statistical correlations and cannot establish causal inferences. Second, this study did not incorporate CSS sampling weights, clustering or stratification design variables due to data‑access constraints, potentially leading to imprecise standard‑error estimation. Third, several important potential confounding variables such as household income, urban‑rural residence, geographic region and occupational profiles could not be incorporated, as these items were not consistently measured or suffered high missing rates across CSS survey waves. Omitted‑variable bias may therefore exist. Fourth, although parallel‑lines tests justified rejecting ordinal‑logistic‑regression and adopting multinomial‑logistic‑regression for four‑category food‑safety‑evaluation outcomes, data sparsity persists for extreme‑outcome categories (“Very unsafe”, “Very safe”). This may compromise parameter‑estimate stability. Fifth, several policy‑related inferences (e.g., the eight‑year trust‑recovery cycle) derive from descriptive interpretation of data trends rather than direct tests of policy‑intervention effects. Some speculative interpretations regarding melamine‑tainted‑milk‑powder crises and media‑exposure impacts are not directly measured within regression models. These readings should therefore be treated as suggestive rather than definitive conclusions and await validation using more granular research designs. Sixth, all food‑safety‑evaluation outcomes rely on self‑reported subjective survey responses, which may be subject to reporting bias, recall bias, and social desirability bias inherent to questionnaire‑based data collection.

## Conclusions

Based upon seven‑wave CSS survey data spanning 2006‑2023, descriptive analysis and multinomial logistic‑regression methods were applied to systematically investigate long‑term shifts and influencing factors shaping public food‑safety evaluation. Key conclusions are presented below.

First, mean public food‑safety‑evaluation scores followed a phased “rise‑fall‑rise” fluctuation pattern from 2006‑2023.

Second, level of social security represented the most robust positive predictor across all survey years (*p* < 0.001), with fluctuating effect sizes over time. Significant interaction effects between survey year and level of social security (*p* < 0.001) demonstrated that spill‑over effects exerted by social governance upon food‑safety evaluation shifted across time and had grown stronger in recent years.

Third, in separate year‑by‑year independent models, educational attainment exhibited stable negative associations with food‑safety evaluation. Effect directions for gender, age and other control variables fluctuated across survey years, demonstrating that their influences were conditioned by period‑specific social contexts.

Fourth, Model diagnostics for year‑stratified models showed that all VIF values were below 1.780, indicating no multicollinearity; the core findings remained statistically significant after Bonferroni correction, and the research conclusions were robust.

Future‑research avenues include incorporating additional macro‑level indicators (e.g., regional food‑safety‑supervision investment, objective sampling‑inspection pass rates), adopting multilevel‑modelling frameworks to analyse interactions between individual‑level perceptions and macroenvironmental factors, and implementing panel‑survey designs to unpack causal mechanisms underlying shifts in food‑safety‑related public perceptions.
